# Overweight and obesity prevalence among public servants in Nadowli district, Ghana, and associated risk factors: a cross-sectional study

**DOI:** 10.1186/s40608-017-0153-5

**Published:** 2017-06-01

**Authors:** Margaret Atuahene, John Kuumuori Ganle, Martin Adjuik, Nana Frema Atuahene, Grace Billi Kampitib

**Affiliations:** 1 Department of Population, Family and Reproductive Health, School of Public Health, University of Ghana, Accra, Ghana; 2 School of Public Health, University of Ghana, Accra, Ghana; 3 Augusta Medical Centre, Waynes borro, VA USA; 4 Ministry of Health, Accra, Ghana

**Keywords:** Overweight, Obesity, Hypertension, Risk factors, Body mass index, Civil servants, Ghana

## Abstract

**Background:**

Globally, overweight and obesity are becoming a mounting concern, impacting negatively on the health of populations especially in low-income settings. However, there is paucity of epidemiological information available in Ghana to support intervention activities. We conducted a study among public servants to estimate overweight/obesity, hypertension and diabetes prevalence and associated risk factors.

**Methods:**

A descriptive cross sectional survey involving 271 purposively sampled public servants aged 20 to 59 years was conducted. We used a structured questionnaire to collect data on eating patterns, risk factors for overweight and obesity, as well as data on socio-demographics and physical activity. Anthropometric measurements were carried out and body mass index (BMI) calculated. Information on blood pressure and diabetes was also gathered. We used descriptive statistical and logistic regression analyses to, respectively, estimate overweight/obesity prevalence, and examine associations between behavioral factors and overweight/obesity and hypertension/diabetes.

**Results:**

The overall hypertension/diabetes, overweight and obesity prevalence were 20, 29.9 and 4.8% respectively. The study found that marital status (*p* < 0.001), leisure time with physical activity and level of physical activity during work (*p* < 0.035) as well as morbidities such as diabetes and hypertension (*p* < 0.012) were significantly associated with BMI. Findings showed no significant relationship between mealtime, eating habits, education, age and body mass index. Even though prevalence of overweight/obesity was higher among respondents who travelled to work by car compared to respondents who used motor bikes or walked, the association between weight status and means of transport was not statistically significant. Both smoking (*p* = 0.730) and alcohol consumption (*p* = 0.109) were not linked to weight status.

**Conclusion:**

Population-based interventions are needed to promote nutritious food selection and consumption, physical activity and healthy life styles. We also recommend that age and gender-specific interventions should be designed and implemented by relevant authorities to promote and support healthy living and healthy-lifestyles at home and in workplaces.

**Electronic supplementary material:**

The online version of this article (doi:10.1186/s40608-017-0153-5) contains supplementary material, which is available to authorized users.

## Background

Increasingly, it is being recognized that a number of low-income countries are currently experiencing an epidemiological transition, a phenomenon that developed countries went through previously [1]. Consequently, populations across several low-income settings, including sub-Saharan African countries, now live under a double burden of infectious and emerging chronic or non-communicable diseases (NCDs) [1]. Globally, NCDs are a major contributor to adult mortality [2–4]. In 2008 for example, NCDs were responsible for some 36 million or 63% of all deaths [2]. It is estimated that low- and middle-income countries accounted for nearly 80% of the 36 million NCDs-related deaths that occurred in 2008 [2].

According to the WHO, up to three quarters of NCDs are preventable through addressing their risk factors [5]. Evidence across the developed world suggest that most NCDs are linked with four particular behavioural risk factors [4]. These are physical inactivity, tobacco use, excessive alcohol use and unhealthy diet [4]. These behavioural risk factors often cause significant key physiological changes, including overweight/obesity, elevated blood pressure, hyperlipidemia, and hyperglycemia. There is evidence in the literature to suggest that raised blood pressure has one of the greatest attributable risk (13%) for death among adults [4]. Other important risk factors for adult mortality are tobacco use (9%), physical inactivity (6%), raised blood glucose (6%), and overweight with obesity 5% [4].

In most low-income settings, the risks of NCDs have been exacerbated by improvements in socio-economic living conditions as well as urbanization [6]. Currently in Africa, the proportion of the urban population that is classified as either overweight or obese is estimated to be 20–50% [7]. Evidence from developed countries suggest that the prevalence of obesity and chronic diseases often tend to be higher among those with low socioeconomic status as well as among those who live in poor neighborhoods. In contrast, evidence from a number of African settings show a strong positive association between high socioeconomic status and obesity [8]. One study for example revealed that improvement in the economic conditions of people has a bearing on health [9]. This study indicated that an increase in average income level was associated with living risky lifestyles such as, consumption of high fat diets, unhealthy snacking, and sedentary work and lifestyles, all of which have bearing on obesity [9]. However, the extent to which this is true across different societies in Africa has not been fully established. Indeed, in the majority of low- and middle-income countries where the burden of NCDs is greatest, the risk factors for chronic NCDs have yet to be adequately characterized. In Ghana for example, public servants over the past 5 years have been migrated onto a single spine salary structure, which has marginally improved on their salaries and ultimately their economic status. Skipping of breakfast and high reliance on meals prepared and consumed away from home among workers form factors that could significantly impact average BMIs [9]. In addition, more sedentary work and general lifestyle among public servants potentially exposes them to chronic NCDs. Indeed, evidence elsewhere have clearly highlighted the problem of NCDs among civil servants. For example, one study that sought to estimate systolic blood pressure (SBP) prevalence, diastolic blood pressure (DBP) prevalence, body mass index (BMI) and fasting plasma glucose (FPG) among 280 formal sector workers in both public and private organizations in Nigeria, found that the prevalence of hypertension was 27.1% (28.4% in males and 22.9% in females) in the study population [10]. The study reported mean SBP and DBP that were significantly higher in females than in males. Obesity prevalence was 13.2% while hypertension prevalence was observed to increase as both age and BMI increased. Diabetes mellitus prevalence in both sexes was similar at 1.5% [10].

Although NCDs-related research is increasingly being conducted in Ghana, including studies on obesity epidemiology in Ghana [11]; variations in obesity by sociodemographic characteristics among Ghanaian adults [12]; healthy lifestyle behaviours among Ghanaian adults [9], and hypertensive target organ damage [13], there are limited population-based studies in Ghana that have specifically characterized overweight and obesity prevalence among public servants - a potentially high-risk population - and the associated risk factors. We are aware of one cross-sectional survey that was conducted among 141 adult male and female staff of one public university in Ghana [14]. Although this study provided important insights on healthy living behaviours, it was limited to only one city and also concentrated on respondents who largely work on the broad area of health. This makes it hard to draw generalizable conclusions in relation to obesity/overweight prevalence among public servants across Ghana. This could potentially hamper the design and implementation of evidence-based interventions. As some researchers have intimated, population-based research and initiatives are needed to identify prevalence and risk factors as well as effective interventions to address overweight and obesity [15–17]. The aim of this study is therefore to estimate overweight/obesity, hypertension and diabetes prevalence, and to determine associated risk factors among public servants in the Nadowli District of Ghana.

## Methods

### Study area

This study was conducted in the Nadowli District of Ghana. Farming accounts for about 85% of the labour force with maize, millet, groundnut and beans as some of the staples and relatively available all year round.

### Study design

This study was cross-sectional in design, and was carried out among public servants in the Nadowli District. Public servants in this study refer to the totality of human resources employed by state or government departments established in accordance with the constitution of Ghana as public service, and those who are on government payroll within the Nadowli District. Women who were public servants but were pregnant at the time of the study were not included. At the time of this study, a total of 775 public servants were employed in a total of 12 public service departments in the district.

We focused on public servants in this study for a number of reasons. First, previous researchers have observed that public servants are an important group of interest when it comes to NCDs because, the lay public generally expect them to be leaders when it comes to living and practicing healthy lifestyles because of their relatively better access to information [14]. At the same time, this group is also at risk of leading sedentary and unhealthy lifestyles partly due to long periods of sitting in work places, and partly because of their relatively better socio-economic status which makes it easier for them to access less physically active modes of transportation such as cars. Second, we focus on this group because less attention has been paid to them in research across Africa and in Ghana, despite the recognition that they could be at heightened risk of NCDs.

### Sample size determination

We determined the appropriate sample size for the study using the Public Service of Creative System survey software (http://www.surveysystem.com). Using the population size of 775, with a confidence level of 95%, a sample size of 257 was obtained. However, 271 questionnaires were administered to cater for data entry errors and non-responses.

### Sampling methods

In terms of sampling, all the 12 public service departments within the district were listed and each department coded. The codes were folded and put in a basket and picked at random until half of them were picked. The subjects were selected through a proportionate random sampling. This sampling technique ensured the inclusion of greater number of respondents from bigger departments. This ensured that our sample was representative of all the different categories of public servants in the district. In order to select a respondent, any person who was present at the time of entering a particular department was interviewed and was asked to call the next available person. This was done in all selected departments until the total sample size was obtained.

### Data collection instruments and procedures

We used structured questionnaires (see Additional file 1) to gather data on respondents’ socio-demographic characteristics such as age, sex, religion, occupation, marital status, health risk behaviours (smoking and alcohol consumption), nutrition knowledge/health and physical activity. Anthropometrics were measured for the calculation of their Body Mass Index. Respondents were weighed wearing light cloths and without shoes. We also measured height of respondents with a suspended microtoise tape to the nearest 1 m without shoes and in minimum clothing standing erect with hands hanging loosely by their sides.

Body mass index (BMI) was calculated as weight in kilograms divided by the square of height in meters. Overweight/obesity was defined as BMI of ≥ 25 kg/m2; and obesity as BMI of ≥ 30 kg/m2.

### Pretesting of data collection instruments

The questionnaire was pretested on selected number of public servants outside the sample area and necessary corrections and adjustments made. The pretest enabled all ambiguities in questionnaire items and responses to be identified and corrected. The pretest also helped to determine how much time was required to complete the questionnaire.

### Data management and statistical analysis

The SPSS statistical software (version 20) was used to enter data, clean the data, and as well edit the data for inconsistencies. All questionnaires that were incomplete or did not meet other quality control tests were not included for analysis. Descriptive statistical analysis such as frequency and percentage distributions were first used to summarize the data as well as describe important characteristics of study respondents. Bivariate and regression analyses were then performed to examine the possible relationship between a number of independent variables such as age, sex, marital status, level of education and leisure time activity, and dependent variables such as BMI and Hypertension and Diabetes status. Statistical significance was held at 95% confidence level and at a *p*-value of < 0.05.

### Operational definitions

#### Hypertension

Measured blood pressure ≥140 mmHg systolic and/or ≥90 mmHg diastolic or self-reported use of drug treatment for hypertension irrespective of measured blood pressure [18].

#### Body Mass Index (BMI)

Normal range BMI (healthy weight) = 18.5–24.9 kg/m2; underweight <18.5 kg/m2; overweight = 25.0–29.9 kg/m2; obesity ≥0.0 kg/m2. These definitions were based on the WHO Classification [19].

#### Physical inactivity

The absence of non-vigorous physical activity for at least 30 min ≥5 days of a week or vigorous physical activity for 20 min in ≥3 days of a week.

#### Tobacco smoking

Civil servants who self-reported current smoking of cigarettes.

#### Alcohol consumption

Consumption of ≥5 drinks of alcohol at one sitting (males); and consumption of ≥4 drinks at one sitting (females).

## Results

In total, 271 public servants were surveyed in this study. Table 1 below shows that 225 (83%) respondents were younger than 40 years and males constituted the majority 171 (63.3%) of the respondents. The average age was 32.7 years (SD = 8.605). About 64% was married and almost all 262 (96.7%) had attained tertiary level of education.

**Table 1:** Prevalence of Overweight and Obesity by Demographic Characteristics

Variable	Overall (%)	Normal (%)	Overweight (%)	Obese (%)	*P* value
Age (years; mean = 32.72,SD = 8.605)					
Younger (<40)	225 (83.0)	159 (89.8)	57 (70.4)	9 (69.2)	0.125
Older (>40)	46 (17.0)	18 (10.2)	24 (29.6)	4 (30.8)	
Sex					
Male	171 (63.3)	119 (67.2)	47 (58.8)	5 (38.5)	0.075
Female	99 (36.7)	58 (32.8)	33 (41.2)	8 (61.5)	
Marital Status					
Married	171 (63.8)	98 (56.3)	61 (75.3)	12 (92.3)	0.001
Unmarried	97 (36.2)	76 (43.7)	20 (24.7)	1 (7.7)	
Religion					
Christianity	230 (85.2)	157 (88.7)	64 (80)	9 (69.2)	0.063
Islam	40 (14.8)	20 (11.3)	16 (20)	4 (30.8)	
Highest Educational level					
Pre-Tertiary	9 (3.3)	5 (2.8)	4 (4.9)	0 (0)	0.537
Tertiary	262 (96.7)	172 (97.2)	77 (95.1)	13 (100)	

The overall overweight and obesity prevalence were 29.9 and 4.8% respectively. It was found that marital status, leisure time and level of physical activity during work as well as morbidities such as diabetes and hypertension were significantly associated with BMI. Obesity was found to be less among those who smoked daily than those who did not. However, respondents who consumed alcohol had high proportion of overweight (55.7) or obesity (63.6%). The obese had less physical activity-sedentary (0.035) than the rest. Participants with history of Diabetes or Hypertension were more obese than those without the conditions. Obesity was highest among those with tertiary education (100%).

Married respondents showed significant (*p* < 0.001) increased risk for overweight (75.3%) and obesity (92.3%) than unmarried respondents (24.7) but no statistically significant association existed between age and BMI. Obesity was also higher among females (61%) than among males (38.5%), albeit the difference was not statistically significant (*p* = 0.075).

Table 2 shows the relationship between weight status and risk factors such as smoking and alcohol consumption. Of all the respondents, 127 (47.9%) consumed alcohol and only 7(2.7%) were smokers. Prevalence of overweight (55.7%) was higher among those who drink alcohol compared to non-drinkers (44.3%). The association between alcohol consumption and weight status was however not statistically significant (*p* = 0.109). Smoking was not also linked to weight status as seen in Table 2 (*p* = 0.730).

**Table 2:** Prevalence of Overweight and Obesity by Smoking and Alcohol (*N* = 265)

Variable	Overall (%)	Normal (%)	Overweight (%)	Obese (%)	*P* value
Alcohol consumption					0.109
Yes	127 (47.9)	76 (43.4)	44 (55.7)	7 (63.6)	
No	138 (52.1)	99 (56.6)	35 (44.3)	4 (36.4)	
Smoking					0.730
Yes	7 (2.7)	5 (2.9)	2 (2.7)	0 (0)	
No	251 (97.3)	167 (97.1)	73 (97.3)	11 (100)	

Table 3 also illustrates the relationship between respondents’ eating patterns and BMI. As shown in Table 3, 150 (55.4%) of respondents eat their most important meal in the afternoon, and 26 (9.6%) in the evening. Of the total respondents, 65 (24.1%) skip breakfast, 136 (50.4%) skip breakfast but not often. Also, 144 (53.1%) eat the last meal of the day between 5:30 pm and 6 pm and 124 (46.9%) after 7 pm. The results did not reveal statistically significant relationship between eating patterns and BMI.

**Table 3:** Prevalence of Overweight and Obesity by Respondents’ Dietary Habit (*N* = 271)

Variable	Overall (%)	Normal (%)	Overweight (%)	Obese (%)	*P* -value
Most important meal					
Morning	95 (35.1)	55 (31.1)	33 (40.7)	7 (53.8)	0.324
Afternoon	150 (55.4)	103 (58.2)	42 (51.9)	5 (38.5)	
Evening	26 (9.6)	19 (10.7)	6 (7.4)	1 (7.7)	
Skipping breakfast					
Yes	65 (24.1)	40 (22.7)	21 (25.9)	4 (30.8)	0.540
No	69 (25.6)	45 (25.6)	23 (28.4)	1 (7.7)	
Sometimes	136 (50.4)	91 (51.7)	37 (45.7)	8 (61.5)	
Last meal of the day					
Around 5:30 pm	144 (53.1)	94 (53.1)	45 (55.6)	5 (38.5)	0.294
After 7 pm	127 (46.9)	83 (46.9)	36 (44.4)	8 (61.5)	

Table 4 however presents data on the distribution of overweight and obesity among respondents who suffered from either diabetes or hypertension. The results indicate that the association between the presence of the conditions and BMI is statistically significant (*p* <0.012).

**Table 4:** Prevalence of Overweight and Obesity among Respondents’ with Hypertention/diabetes (*N* = 265)

Variable	Overall (%)	Normal (%)	Overweight (%)	Obese (%)	*P* value
Condition suffered from					
Hypertension or Diabetes	20 (7.5)	7 (4)	11 (13.9)	2 (18.2)	0.012
None	245 (92.5)	168 (96)	68 (86.1)	9 (81.8)	

Table 5 indicates that 168 (63.6%) of respondents did not exercise at all, and 13 (4.9%) exercised but not often. The results revealed significant association between weight status and leisure time activities (*p* < 0.000). Some 212 (78.2%) of respondents were involved in jobs that involved physical activity and 59 (21.8%) were involved more in sedentary work, that is, jobs that involved sitting most of the time. Majority, 212 (78.8%) of respondents commuted to work by car or by public transport and only 57 (21.2%) either walk or used bicycle as their means of transport. No significant association between weight status and means of transport was however detected, even though prevalence of overweight 61 (75.3%) and obesity 11 (84.6%) were quite high among those who travelled to work by car than in those who used motor bikes or walked.

**Table 5:** Prevalence of Overweight and Obesity by Leisure time and Physical Activity (*N* = 271)

Variable	Overall (%)	Normal (%)	Overweight (%)	Obese (%)	*P*-value
Exercise					
don’t exercise	168 (63.6)	111 (64.5)	51 (63.8)	6 (50)	0.664
Exercise often	83 (31.4)	52 (30.2)	25 (31.2)	6 (50)	
Not too often	13 (4.9)	9 (5.2)	4 (5)	0 (0)	
Leisure time					
Involves physical activity	60 (22.2)	25 (14.2)	29 (35.8)	6 (46.2)	0.000
No physical activity involves	210 (77.8)	151 (85.8)	52 (64.2)	7 (53.8)	
Level of physical activity at work					
Sedentary	59 (21.8)	38 (21.5)	16 (19.8)	5 (38.5)	0.035
Non sedentary	212 (78.2)	139 (78.5)	65 (80.2)	8 (61.5)	
Means of transport					
Car/public transport/motorbike	212 (78.8)	140 (80)	61 (75.3)	11 (84.6)	0.605
Walking/Cycling	57 (21.2)	35 (20)	20 (24.7)	2 (15.4)	

## Discussion

This study examined the prevalence of overweight/obesity, hypertension and diabetes and associated risk factors among public servants in Ghana. The study revealed a number of findings that should not be ignored.

### Socio-demographical status and obesity

Both overweight and obesity were detected among respondents of different socio-demographic backgrounds. Overall, overweight and obesity prevalence were 29.9 and 4.8% respectively. The prevalence of obesity in our study population is higher than the 4.6% prevalence reported among the general adult population in Ghana by [12]. Together with previous findings from other African urban settings such as Nigeria [10, 20], the findings in this study suggests that public servants are indeed one sub-population at particular risk of overweight and obesity, and related consequences of hypertension and diabetes. This result is however counter-intuitive because public servants generally are educated and also have greater opportunities for obtaining healthy behavioural change information. Therefore, one would expect better outcomes and lifestyle indicators for public servants than for the general public [14]. However, our results that both overweight and obesity levels among respondents are higher than the general population in Ghana clearly do not support the assumption above. Rather, the finding lends support to previous research which suggested statistically significant association between obesity and high socioeconomic status (SES) [8]. Indeed, although the link between SES and overweigh/obesity was not directly investigated in our study, it is plausible to think that increased SES among public servants is a factor contributing to the relatively high levels of overweight and obesity prevalence among our study’s respondents. As noted earlier, salaries of public servants over the past 5 years in Ghana have marginally improved. This has ultimately improved their economic status above the general population. In addition to the risks associated with sedentary work and general lifestyle among public servants, consumption of processed foods, unhealthy snacking, consumption of high fat diets away from home are all behaviours that are associated with increasing purchasing power particularly in low-income settings [20]. Coupled with the fact that several of the public servants surveyed in this study were not regularly engaged in any vigorous physical activity or rode cars or public buses to work, our findings suggest the need for promotive health interventions (e.g. healthy eating) targeted at public servants not only in our study context of Ghana but also in other low-income settings such as Nigeria where similar high levels of overweight and obesity have been reported among public servants.

### Hypertension/diabetes associated with obesity

The prevalence of 20% in hypertension/diabetes obtained from this study compares favourably with the results of one Nigerian study which reported 27.1% [10]. Overweight and obesity are known risk factors for hypertension and diabetes. This is partly because increases in body weight can lead to increases in insulin resistance as well as defects in insulin secretion. Also, the risk of developing Type 2 diabetes and/or hypertension is increased with increases in overweight and/or obesity [21]. As indicated, respondents who had either diabetes or hypertension were more likely to be overweight and obese compared to those who were not hypertensive or diabetic and was significantly related with BMI (P <0.012). Indeed, hypertension and obesity have been known to be positively related [14]. Findings indicated abdominal obesity in particular played a greater role in elevated blood pressure. In this study however, no association was found between these behavioural factors and selected NCDs. This could partly be explained by the cross- sectional nature of the study. It could also be due to the fact that the behavioural factors examined in this study were self-reported by respondents. Therefore, the possibility of exaggeration of socially acceptable practices by respondents may have affected the relationship.

### Smoking and alcohol consumption

This study also examined the link between smoking, alcohol intake and weight status. No association was found between smoking and weight status (p > 0.730). Previous studies have however found alcohol consumption to be positively correlated with BMI in men or in both sexes [22, 23]. Other recent studies have however indicated that the association between alcohol consumption and BMI could be explained by differences in consumption patterns [24–26]. For example, among adults, the amount or intensity of drinking per drinking occasion has been found to be positively correlated with BMI [24, 25]. At the same time, the frequency of drinking has been found to be negatively correlated with BMI [26]. This latter finding would suggest that frequent light drinking might offer a protective effect or benefits. Also no statistically significant association was found between alcohol consumption and BMI (p > 0.109). The lack of association could partly be due to the relatively small proportion of alcohol drinkers and smokers in our study. Again current smokers have lower BMI than former smokers and this study involved only current smokers.

### Leisure time and physical activity

Increasingly, a number of researchers are recognizing that physical inactivity arising from sedentary forms of work and lifestyles, changing modes of transportation, and increasing urbanization, is a major factor contributing to obesity [2, 4]. Among the respondents in this study, leisure time activity, which was apparently dominated by sedentary behaviours such as long sitting hours watching television and drinking with friends, was positively and significantly associated with BMI (P < 0.000). This finding supports earlier results by [15] which suggested that sedentary behaviours were associated positively with obesity. The result is also consistent with another study among subjects from two urban and one rural community, which found high levels of overweight and obesity among subjects who were sedentary and engaged in light activity [10]. The lack of physical activity among the public servants surveyed in this study could partly be one of the factors responsible for the high overweight prevalence. In terms of policy response, our findings here suggest the need for promotive health interventions targeted at public servants such as more physical activities such as playing sports and regular exercising. This recommendation is indeed in line with the recent WHO’s recommendations related to the prevention of NCDs in the workplace through diet and physical activity’ [27].

### Study limitation

This study was a cross- sectional study which could lead to limited study conclusion between risk factors and overweight. Again BMI alone is not always an accurate predictor of body fat or distribution of adiposity, particularly as it may overestimate body fat in muscular individuals and underestimate in older subjects because of muscle mass loss.

## Conclusions

Risk factors for overweight and obesity identified in this study had significant association with social support such as marital status, hypertension/diabetes, leisure time activities and sedentary lifestyle. Population-based interventions should be encouraged to promote workplace physical activity, healthy food selection, leisure time activities and social support. Also, workplace and community-based smoking cessation programs, together with interventions to encourage responsible alcohol-intake, should be added to interventions in the work environment. Collaboration on screening of departmental workers for various non-communicable diseases and counseling on behavioral risk factors should also be promoted.

## Additional file

Additional file 1: Study Questionnaire. Questionnaire for studying prevalence of overweight/obesity among public servants in Ghana. (DOC 80 kb)

## Abbreviations

BMI: Body mass index
DBP: Diastolic blood pressure
FPG: Fasting plasma glucose
NCDs: Non-communicable diseases
SBP: Systolic blood pressure
SES: Socioeconomic status
WHO: World Health Organization
