# Supplementary material for: Overweight and obesity prevalence among public servants in Nadowli district, Ghana, and associated risk factors: a cross-sectional study
Source: BMC Obes. 2017 Jun 1;4:15. doi: 10.1186/s40608-017-0153-5 (PMC5452290; doi:10.1186/s40608-017-0153-5)
Supplement: Additional file 1: — Study Questionnaire. Questionnaire for studying prevalence of overweight/obesity among public servants in Ghana. (DOC 80 kb) [file 40608_2017_153_MOESM1_ESM.doc]

**Overweight and Obesity Prevalence among Public Servants in Nadowli district, Ghana, and associated risk factors: a cross-sectional study**

Section A: Demographic characteristics/Background Information (please tick as appropriate)

| **NO.** | **QUESTIONS & FILTERS** | **CODING CATEGORIES** | **GO TO** |
| --- | --- | --- | --- |
| 1 | Which Institution/place do you work? | ……………………………………. |  |
| 2 | What is your profession? | ……………………………………. |  |
| 3 | How old are you?  **PROBE: how old were you at your last birthday** | |  | | --- |   Respondent’s Age |  |
| 4 | What is your Sex? | Male………………………………. [ 1 ]­  Female………………….………… [ 2 ]­ |  |
| 5 | What is your current marital status? | Living with partner………………… [1 ]  Married……...………………………[ 2 ]  Widowed……………………………[ 3 ]  Divorced/separated…………………[ 4 ]  Single……………………….……… [ 5 ] |  |
| 6 | Which Religion do you associate yourself with? | Christianity ………………….………[ 1 ]  Islam ………………………...………[ 2 ]  Traditional………………..………….[ 3]  Other (specify)……………….……...[ 4 ] |  |
| 7 | Have you ever attended school? | Yes………………….……….………[ 1 ]  No…………..…………….…………[ 2 ] | **9** |
| 8 | What is the highest level you attained? | Primary………………….……..……[ 1 ]  Middle/JHS………………….....……[ 2 ]  SHS………………..………………. .[ 3 ]  Tertiary……………….……….…..... [ 4 ] |  |

Section B: Knowledge of public servants on diet, health and disease.

| **NO.** | **QUESTIONS & FILTERS** | **CODING CATEGORIES** | **GO TO** |
| --- | --- | --- | --- |
| 9 | Which of these have an influence on weight gain? | Eating high fat diets………………....[ 1 ] ­  Increased alcohol consumption ...…. [ 2 ]­  Lack of physical activity….………. [ 3 ]­  Lack of appetite….…………….…… [ 4 ]­  Late night eating…………………..... [ 5 ]  Others (specify)……………………..[ 6 ] |  |
| 10 | In your opinion, does alcohol consumption contribute to overweight & obesity? | Yes………………….……….………[ 1 ]  No…………..…………….…………[ 2 ]  Don’t know...………………………..[ 3 ] |  |
| 11 | In your opinion does smoking contribute to overweight & obesity? | Yes………………….……….………[ 1 ]  No…………..…………….…………[ 2 ]  Don’t know ………………………...[ 3 ] |  |
| 12 | Which of the following do you think are major health benefits of engaging in physical activity? | Reduce stress and improve general wellbeing............................................ .[ 1 ]  Stimulate weight loss……...……….....[ 2 ]  Lowers blood pressure…..…..………..[ 3 ]  Builds up muscles and increase weight [ 4  Others (specify )………………………[ 5 ] |  |
| 13 | Which of these do you think are associated with low intake of fruits and vegetables? | hypertension………………………….[ 1 ]  some cancers.………..……………….[ 3 ]  Diabetes ………..………...………….[ 4 ] |  |
| 14 | What type/kind of food in your opinion can make you obese? | Khebabs and stews……………...……[ 1 ]  Kenkey and fish………………...……[ 2 ]  Soft drinks …………………… ……...[ 3 ]  Others (specify)……………………....[ 4 ] |  |
| 15 | If you have to eat in the day when do you think you will eat the most important meal? | Morning …………..…………………[ 1 ]  Afternoon....…………………….…...[ 2 ]  Evening...…..…………………..……[ 3 ]  Not Sure………..…………………….[ 4 ] |  |
| 16 | Which of these conditions do you think can result from obesity? | Diabetes….………………...……….....[ 1 ]  Difficulty in breathing…...………..... [ 2 ]  Lung Cancer………....…………….....[ 3 ] |  |

**Section C: T**o determine the distribution of Body Mass Index (BMI) among public servants in the Nadowli District

| **NO.** | **QUESTIONS & FILTERS** | **CODING CATEGORIES** | **GO TO** |
| --- | --- | --- | --- |
| 17 | Could you permit me to take your height and weight? | Height in cm   |  | | --- | |  |   Weight in kg |  |
|  | Body Mass Index | Weight in kg/height in cm square   \|  \| \| --- \| |  |

Section D: Factors that predispose public servants to overweight and obesity

| **NO.** | **QUESTIONS & FILTERS** | **CODING CATEGORIES** | **GO TO** |
| --- | --- | --- | --- |
| 18 | Briefly describe your activities on a typical working day | Desk officer ………………………... [ 1 ]  A field worker ………………………[ 2 ]  A Driver …………………………….[ 3 ] work involve standing and moving round…………………………………[ 4 ]  Others (specify)……………………...[ 5 ] |  |
| 19 | In an average working day how many hours do you spend doing these activities at work? | 3-4 hours.…………………….……...[ 1 ]­  6 hours...………………….…………[ 2 ]­  All day…………………….……… [ 3 ]­  Others (specify)...……………….… [ 4 ]­ |  |
| 20 | Do you often skip breakfast? | Yes………………….……….………[ 1 ]  No…………..…………….…………[ 2 ]  Sometimes………….…….…………[ 3 ] |  |
| 21 | Do you usually eat at restaurants and canteens/ chop bars? | Yes………………….……….………[ 1 ]  No…………..…………….…………[ 2 ] | **24 23** |
| 22 | If yes, How many times in a week? | Once in a week……..…….…………[ 1 ]  Twice in a week……...……………...[ 2 ]  Every day of the week…..…..………[ 3 ]  Others (specify)……………………..[ 4 ] |  |
| 23 | When do you usually take the last meal of the day? | At 5:30 pm……………..………........[ 1 ]  Between 6 and 7 pm………………...[ 2 ]  Between 7 and 8 pm………………...[ 3 ]  Others (specify)…………………….[ 4 ] |  |
| 24 | Which of these transports do you often use to work and for other activities? | By car……..................................……[ 1 ]  Public transport………………...……[ 2 ]  Walking……………………………. .[ 3 ]  Motor bike…………………………. .[ 4 ]  Others (specify)……………………...[ 5 ] |  |
| 25 | How do you spend your leisure time? | Cleaning the house ………………….[ 1 ]  Gardening……...……………..……..[ 2 ]  Reading and watching TV…..………[ 3 ]  Drinking with friends………………. [ 4 ]  Other (specify)………..……………..[ 96 ] |  |
| 26 | How often do you engage in this activity? | Everyday………………………..........[ 1 ]  Once in a week……………… ….…..[2 ]  Twice in a week……………..…..…..[ 3 ]  Others (specify)……………………..[ 4 ] |  |
| 27 | Do you take alcoholic beverages? | Yes………………….……….………[ 1 ]  No…………..…………….…………[ 2 ] | **2929** |
| 28 | If yes, on an average how many bottles of beer/tots of gin/calabashes of pito do you take in a day? | One a day……..…….…………..……[ 1 ]  Two or more in a day.…………….....[ 2 ]  Others specify...…..…..……………..[ 3 ] |  |
| 29 | Do you smoke? | Yes………………….……….………[ 1 ]  No…………..…………….…………[ 2 ] | **3131** |
| 30 | If yes what do you smoke? | Refined cigarette...…..…….……...….[ 1 ]  Tobacco in a pipe……..……...……...[ 2 ]  Tobacco wrapped in paper...…...........[ 3 ]  Indian hemp…………………………[ 4 ] |  |
| 31 | Which of these conditions do you suffer from? | Diabetes……..…….………............…[ 1 ]  Hypertension……...…………............[ 2 ]  None…..…..............................………[ 3 ] | **32**  **33** |
| 32 | If (1), When was the last time you checked your blood pressure | A week ago……..…….…….......……[ 1 ]  A month ago……...……….....……....[ 2 ]  More than a month…..…..……......…[ 3 ]  Never checked…..…..…............……[ 4 ] |  |
| 33 | If (2), When was the last time you checked your blood sugar | A week ago……..…….……......…….[ 1 ]  A month ago……...………….....…....[ 2 ]  More than a month…..…........………[ 3 ]  Never checked…..…..….............……[ 4 ] |  |
| 34 | How often do you do exercise | Daily ……………………………… [ 1 ]  Twice in a week…………………….. [ 2 ]  Three times in a week………………..[ 3 ]  Others (specify)……………………... [ 4 ] |  |
| 35 | What is the duration of exercise each time | 30 minutes each time ………………..[ 1 ]  1 hour each time ……………………..[ 2 ]  Between 1 and 2 hours ………………[ 3 ]  Others (specify)………………………[ 4 ] |  |
| **Thank You – The End** | | | |
